# Association between inflammatory biomarkers and venous thromboembolism: a systematic review and meta-analysis

**DOI:** 10.1186/s12959-023-00526-y

**Published:** 2023-07-31

**Authors:** Jiayue Ding, Xuanye Yue, Xiaobing Tian, Zhangyuan Liao, Ran Meng, Ming Zou

**Affiliations:** 1 Department of Neurology, Tianjin Medical University General Hospital, Tianjin, 300052 China; 2 Department of Interventional Neurology, Tianjin Medical University General Hospital, Tianjin, 300052 China; 3 Department of Neurology, Xuanwu Hospital, Capital Medical University, Beijing, 100053 China

**Keywords:** Venous thromboembolism, Inflammation, Pulmonary embolism, Deep vein thrombosis, Cerebral venous thrombosis

## Abstract

**Background:**

Venous thromboembolism (VTE) is a common thrombotic vascular disease that has a significant impact on people’s well-being and quality of life. A plethora of clinical studies explore the relationship between inflammatory biomarkers and VTE but yield conflicting results. This article proposed to pool these studies to draw a more convincing conclusion.

**Methods:**

We searched several databases for studies before April 2023. Available data was processed using Stata software (version 15.0 SE) and R (version 4.1.2). This meta-analysis has been registered in PROSPERO (CRD42022321815). The VTE in this review encompassed pulmonary embolism, deep vein thrombosis, and cerebral venous thrombosis.

**Results:**

A total of 25 articles were finally involved in this study. Our results revealed that higher levels of high-sensitivity C-reactive protein (hs-CRP, MD, 0.63, 95%CI, 0.21—1.05) and C-reactive protein (CRP)> 3ug/ml (OR, 1.52, 95%CI, 1.18—1.96) might be regarded as risk factors for future VTE occurrence. The elevated levels of monocyte (MD, 0.03, 95%CI, 0.00—0.05), hs-CRP (0.85, 0.61—1.08), CRP (0.66, 0.20—1.13) and IL-6 (0.47, 0.25—0.70) might represent the previous VTE; a series of markers such as white blood cell (1.43, 0.88—1.98), neutrophil (1.79, 1.02—2.56), monocyte (0.17, 0.14—0.21), hs-CRP (3.72, 1.45—5.99), IL−6 (5.99, 4.52—7.46), platelet-lymphocyte ratio (33.1, 24.45—41.78) and neutrophil-lymphocyte ratio (1.34, 0.95—1.73) increased during the acute phase of VTE.

**Conclusions:**

In general, activated inflammatory biomarkers might not only be correlated with an increased risk of VTE, but may also give a hint of the occurrence of VTE in clinical settings.

**Supplementary Information:**

The online version contains supplementary material available at 10.1186/s12959-023-00526-y.

## Background

Venous thromboembolism (VTE) is a common thrombotic vascular disease that has a significant impact on people’s well-being and quality of life. It occurs in approximately 1/1000 people per year [1]. The pathogenesis of the initiation and development of VTE has garnered great attention in recent years. Inflammation is considered to play an important role in this process [2, 3]. Similar to arterial ischemia, venous congestion can cause an inflammatory response exacerbating tissue injury [4, 5]. Leukocytes and inflammatory factors are identified as the major culprits in the formation and development of venous thromboses, which further impact the prognosis of VTE [4–6]. Briefly, there are three major pathways involved in inflammatory reactions of VTE: (1) Neutrophils extrude neutrophil extracellular traps (NETs) that can directly activate factor XII (FXII), bind to von willebrand factor (vWF), trigger platelet recruitment and increase the concentration of enzymes such as neutrophil elastase and myeloperoxidase, to initiate the intrinsic coagulant pathway; (2) monocytes release microparticles containing activated tissue factor (TF) at sites of pathogen exposure; extrinsic coagulant pathway is triggered finally assisting NETs; (3) pro-inflammatory cytokines, especially IL-6 can modulate the inflammatory process to affect the coagulant system and fibrinolytic system; adhesion molecules, such as P-selectin, can initiate rolling leukocytes adhering to activated platelets and endothelial cells, leading to neutrophil migration, NET formation, and TF-bearing microparticles release, so as to further amplify the inflammatory reactions in venous thrombus formation and evolution [7–9]. In addition, inflammatory diseases such as systemic lupus erythematosus, Behçet’s disease, and inflammatory bowel disease, that cause complications with activated inflammatory responses in the venous system, serve as risk factors for VTE [10]. Therefore, inflammation triggered by VTE, acts as promoters of the occurrence and development of VTE in turn.

Inspired by the results of the animal experiments, some researchers tested their hypothesis with the clinical assay. A plethora of clinical studies explore the fluctuations of inflammatory markers in patients with VTE but yield conflicting results. Fox et al. first conducted a systematic review regarding the relationship between inflammation and VTE in 2005; however, they did not obtain convincing evidence to support their hypothesis due to the small sample size [3]. Whether inflammatory markers are capable of characterizing VTE formation and progression in clinical settings is still in doubt. Given the large number of clinical studies published in recent years, a secondary analysis is warranted to pool these studies to draw a more convincing conclusion.

Cerebral venous thrombosis (CVT) is considered a life-threatening subtype of stroke in the Department of Neurology. Inflammation plays an important role in CVT formation and development, as demonstrated by both animal experiments and clinical studies [11]. The well-known conventional VTE—pulmonary embolism (PE) and deep vein thrombosis (DVT) features an inflammatory response similar to that of CVT. Therefore, we proposed to regard CVT, PE, and DVT as VTE in this study to comprehensively represent VTE-related inflammation.

In this study, we recapitulate a precise overview of current published articles relating to the association of inflammatory markers with VTE, including PE, DVT and CVT. We will investigate the subclinical presentations of inflammatory markers in the patients with future VTE, previous VTE and newly diagnosed VTE. The results of our study will facilitate understanding of inflammation in VTE, and may also provide novel insights into the diagnostic and therapeutic paradigms for VTE in the future.

## Methods

This meta-analysis has been registered in PROSPERO (International Prospective Register of Systematic Review) with the number of CRD42022321815 and was performed in accordance with the Preferred Reporting Items [12].

### Search strategies

Several literature databases were searched in this study, namely, PubMed and Embase for publications that were published prior to April 2023 (a search strategy per database is shown in Table S1). The references of retrieved articles were thoroughly reviewed subsequently for additional reports that we might have miss out in our search. Prospective cohort studies, retrospective case-control studies and cross-sectional studies would be included.

### Study selection

The enrolled studies should meet the following criteria: (1) patients with a definitive diagnosis of PE, DVT, and CVT; (2) inflammatory markers expressed as means ± standard deviate (SD) or median (interquartile range, IQR); (3) comparisons between PE, DVT, CVT, and health controls. Exclusion criteria included: (1) patients with complications at other sites due to venous thrombosis, such as renal veins and mesenteric veins; (2) inflammatory markers presented with median (range/95% confidence interval, 95%CI), mean (range/IQR/95%CI), odds ratio (OR) and relative risk (RR); (3) VTE patients with transient risk factors (such as recent surgery, trauma, fracture, estrogen therapy before VTE diagnosed).

### Data extraction

The following information was retrieved for each article: study design, demographics, inflammatory markers, blood sample collection time and VTE diagnosis time. According to the objectives of the involved studies, we classified these studies into Part I (inflammatory markers as risk factors for VTE events), Part II (inflammatory markers in patients with previous VTE) and Part III (inflammatory markers in patients with newly diagnosed VTE). The available markers included white blood cells (WBC), platelet, neutrophils, lymphocytes, monocytes, platelet-lymphocyte ratio (PLR), neutrophil-lymphocyte ratio (NLR), high-sensitivity C-reactive protein (hs-CRP), C-reactive protein (CRP) and interleukin-6 (IL-6). The mean (SD) was used for the pooled analysis, and the median (IQR) would be conversed with the mean (SD) for the meta-analysis in this study. Data was collected by four reviewers (JY-Ding, XB-Tian, ZY-Liao and XY-Yue), and if inconsistency existed between the two reviewers, the two other reviewers would re-examine the data and made a final decision based upon the majority.

### Statistical analysis

Available data was processed using Stata software (version 15.0 SE) and R (version 4.1.2) in this study. The remaining variables were presented with mean difference (MD), 95% confidence interval (CI) and dichotomous variables were expressed as OR, 95%CI to reach the outcome statistics. Estimating the sample mean (SD) from median (IQR) was done by referring to the methods provided by Luo et al. and Wan et al. [13, 14] We excluded studies presenting other data findings because we considered that conversing from them to mean (SD) was absolutely inaccurate. A funnel plot was used to assess publication bias (RevMan 5.3). Chi-Square test was used to assess the heterogeneity of data. Pooled analysis was performed with fixed-effects model using Mantel-Haenszel method when the heterogeneity was expected to be available (I^2^ < 50%). While, the random-effects model computed by the DerSimonian-Laird method was conducted (I^2^ ≥ 50%). P-value < 0.05 were considered statistically significant (As for MD, 95%CI, the upper limit of 95%CI < 0 or the lower limit > 0 indicate P-value < 0.05; as for OR, 95%CI, the upper limit of 95%CI < 1 or the lower limit > 1 indicate P-value < 0.05). After all available studies were grouped together, sensitivity analysis was performed to reduce heterogeneity. Studies without a definitive blood sample collection time or VTE diagnosis time would be ruled out, and the rest of the studies were coined as ‘Model 1’; the studies using median (IQR) for analysis were removed, and the remaining studies were renamed ‘Model 2’. Data from Model 1 and Model 2 were pooled, respectively. We carried out a network meta-analysis using Bayesian hierarchical models (“gemtc” package of R) to perform pairwise comparisons among the patients with PE, DVT, CVT and controls.

## Results

### Search results and the characteristics of the involved studies

The search strategy identified 247 articles with adopted title published before April 2023. After screening process, 25 articles that followed our study selection criteria remained in this study [13–37]. A screening process flow chart is shown in Figure S1. The afflicted cohorts involved, included patients with PE and/or patients with DVT who were called patients with PE/DVT. The methods of Zhou et al. (2015) and Zhou et al. (2014) were used to derive one sample. We used the data from Zhou et al. (2015) for the pooled analysis in this study [35, 38]. Both Folsom et al. and Tsai et al. extracted data from “the Atherosclerosis Risk in Communities (ARIC)” cohort [15, 16]. However, Tsai et al. analyzed the ARIC cohort data collected from 1987 to 1989, and Folsom et al. examined the cohort data from 1990 to 1992. Besides, Tsai et al. were also involved in the established cohort of “the Cardiovascular Health Study (CHS)”. Zacho et al. enrolled two cross-sectional studies named “the Copenhagen City Heart Study (CCHS)” and “the Copenhagen General Population Study (CGPS)” [13]. Although the CGPS is a cross-sectional study, they included all participants regardless of whether VTE occurred before or after CRP measurement. It cannot verify the predictive role of CRP on the occurrence of VTE, thus this cohort was finally excluded from this study. Artoni et al. compared the markers among patients with PE/DVT, CVT, and controls [18]. Zakai et al. performed retrospective studies from September 2000 to August 2002 and January 2002 to June 2009 respectively, with the same inclusion and exclusion criteria [27, 30]. The data from these two cohorts were included in this study. The mean (SD) of the markers in patients with PE/DVT and CVT was combined and the total mean (SD) was obtained for analysis in this study. A summary of the characteristics of the involved studies is shown in Table 1.

**Table 1:** The characteristics of the involved studies

Author, year	Design (database)	VTE cases, n.	Controls, n.	Blood indexes measured time
**Part I**				
Tsai, 2002	Cross-sectional study (CHS and ARIC)	General populations with PE/DVT (159)	General populations without PE/DVT (19,078)	Blood samples were collected at baseline and participants were followed up to 7.8 years (median) for VTE occurrence.
Zacho, 2010	One prospective and one cross-sectional study (CCHS and CGPS)	General populations with PE/DVT (335)	General populations without PE/DVT (7938)	CCHS: CRP levels were measured in participants at the 1991 to 1994 examination and subsequently followed up to 16 years for VTE occurrence. CGPS: CRP levels were detected in participants from 1976 to 2007 irrespective of whether the VTE occurred before or after the CRP measurement.
Olson, 2014	Prospective longitudinal cohort study (REGARDS)	General populations with PE/DVT (268)	General populations without PE/DVT (27,539)	Blood samples were collected at baseline and participants were followed up to 4.9 years for VTE occurrence.
Folsom, 2018	Cross-sectional study (ARIC)	General populations with PE/DVT (527)	General populations without PE/DVT (9317)	Blood samples were collected at baseline and the VTE follow-up was over a median 17.6 years
**Part II**				
Vormittag, 2005	Retrospective case-control study	Patients with unprovoked PE/DVT (214)	Healthy individuals (104)	Blood samples were collected at study inclusion, and patients were enrolled in the study at least three months after VTE.
Luxembourg, 2009	Retrospective case-control study (MAISTHRO)	Patients with unprovoked PE/DVT (101)	Healthy individuals (202)	The temporal distance range between VTE confirmation and the blood sample collect was 3 months to 5.5 years.
Matos, 2011	Retrospective case-control study	Patients with VTE and treated with oral anticoagulant for at least 6 months^*^ (119)	Healthy individuals (126)	Blood sample collection took place at least 1 month after the discontinuation of oral anticoagulant and > 7 months after the event of VTE.
Poredos, 2011	Retrospective case-control study	Patients with unprovoked DVT (49)	Healthy individuals (48)	Blood samples were collected 2–4 months after DVT diagnosis.
Rattazzi, 2013	Retrospective case-control study	Inpatients with previous PE/DVT (240)	Cases without previous PE/DVT (240)	The blood sample collection was made up 68.3(range 4−156) months after VTE event.
Yang, 2015	Retrospective case-control study	Patients with solid tumor and PE/DVT (76)	Patients with solid tumor but without PE/DVT (97)	Blood samples were obtained no more than 2-month after VTE diagnosis.
Artoni, 2018	Retrospective case-control study	Patients with VTE^*^ (586)	Patients’ partners without VTE (299)	All samples were collected at least 3 months after VTE occurrences.
**Part III**				
Lowe, 2000^#^	Retrospective case-control	Women with unprovoked VTE^#^ (49)	Women without VTE (100)	NA
Reiter, 2003	Prospective pilot	Patients with DVT (37)	Patients without DVT (63)	Blood samples and duplex sonography were made at admission.
Zakai, 2004	Retrospective case-control	Patients with PE/DVT after diagnosis of general medicine, nephrology, oncology and cardiology (65)	Patients without PE/DVT (123)	Blood sample collection and VTE diagnosis took place during hospitalization
Ramacciotti, 2011	Retrospective case-control	Patients with acute DVT (62)	Patients without DVT (116), healthy individuals (30)	Blood samples were obtained before anticoagulant therapy was initiated when DVT was diagnosed.
KAMIŞLI, 2012	Retrospective case-control	Patients with CVT (35)	Healthy individuals (27)	Blood samples were obtained before CVT diagnosis
Zakai, 2013	Retrospective case-control (MITH)	Inpatients with PE/DVT after diagnosis of general medicine, nephrology, oncology and cardiology (299)	Inpatients without VTE (601)	Blood samples were collected at admission, and VTE was confirmed in hospitalization.
Bakirci, 2015	Retrospective case-control	Inpatients with PE/DVT (77)	Healthy individuals (34)	Blood samples were drawn at the first day of VTE diagnosis.
Kurtipek, 2015	Retrospective case-control	Patients with acute PE (71)	Healthy individuals (77)	Blood samples were drawn and definite diagnosis of acute PTE was confirmed during hospitalization.
Zhou, 2015^#^	Retrospective case-control	Inpatients with PE/DVT (68)	Healthy individuals (82)	NA
Akboga, 2017^#^	Retrospective case-control	Patients with CVT (80)	Individuals without CVT (197)	Blood samples were collected at admission and the time of CVT diagnosis was unknown.
Ming, 2018	Retrospective case-control	Patients with unprovoked acute DVT (115)	Healthy individuals (105)	DVT was newly diagnosed and blood samples were collected at admission.
Wang 2018	Retrospective case-control	Inpatients with CVT (95)	Inpatients without CVT (41)	CVT was newly diagnosed and blood samples were obtained at admission.
Farah, 2019	Retrospective case-control	Inpatients with PE/DVT (272)	Inpatients without PE/DVT (55)	Blood samples and duplex sonography were made at hospitalization.
Tekeşin, 2019	Prospective case-control	Inpatients with CVT (36)	General populations without CVT (40)	Blood sample collection and VTE diagnosis took place at admission

^*^VTE comprises of DVT, PE and CVT. ^#^Studies miss time of VTE diagnosis and blood sample collection.

### Characteristics of the involved populations

The objectives and chronological orders of VTE diagnosis and sample collection in the involved studies are diverse, leading to different interpretations of the results (Table 1).

Part I: Two prospective longitudinal studies and three community-based cross-sectional studies in four publications observe general populations for a long time, in which the samples are collected at enrollment and the participants are followed for longer than 4.6–16 years for the occurrence of VTE [13–16]. These studies included established cohorts from CCHS, CGPS, the REasons for Geographic and Racial Differences in Stroke (REGARDS), CHS, and ARIC. Persons with cancer are excluded in two studies (Olson et al. and Tsai et al.), while the remaining two (Folsom et al. and Zacho et al.) have no concomitant disease restriction.

Part II: Seven retrospective studies recruit patients with and without previous VTE or health populations, in which the time distance between the diagnosis of previous VTE and sample collection is varied from no more than 2 months to at least 7 months [17–23]. One study (Yang et al.) is carried out in the setting of a solid tumor, 3 studies (Poredos et al., Vormittag et al. and Luxembourg et al.) focus only on unprovoked VTE, and 1 study (Matos et al.) includes patients with oral anticoagulant treated for at least 6 months.

Part III: Two prospective studies and nine retrospective studies enrolled patients with and without newly diagnosed VTE or health populations, in which blood samples are obtained at admission or during hospitalization [24–37]. One study (Ming et al.) only investigates patients with unprovoked DVT. Zakai et al. only included patients after diagnosis by medicine, nephrology, oncology, and cardiology departments. There are three studies that do not have a definitive time for the diagnosis of VTE and collection of blood samples, and we put them in Part III temporarily [35–37]. Next, we performed a sensitivity analysis by removing these studies to retest our conclusions.

### Part I: inflammatory markers as risk factors of VTE

**Fig. 1:**
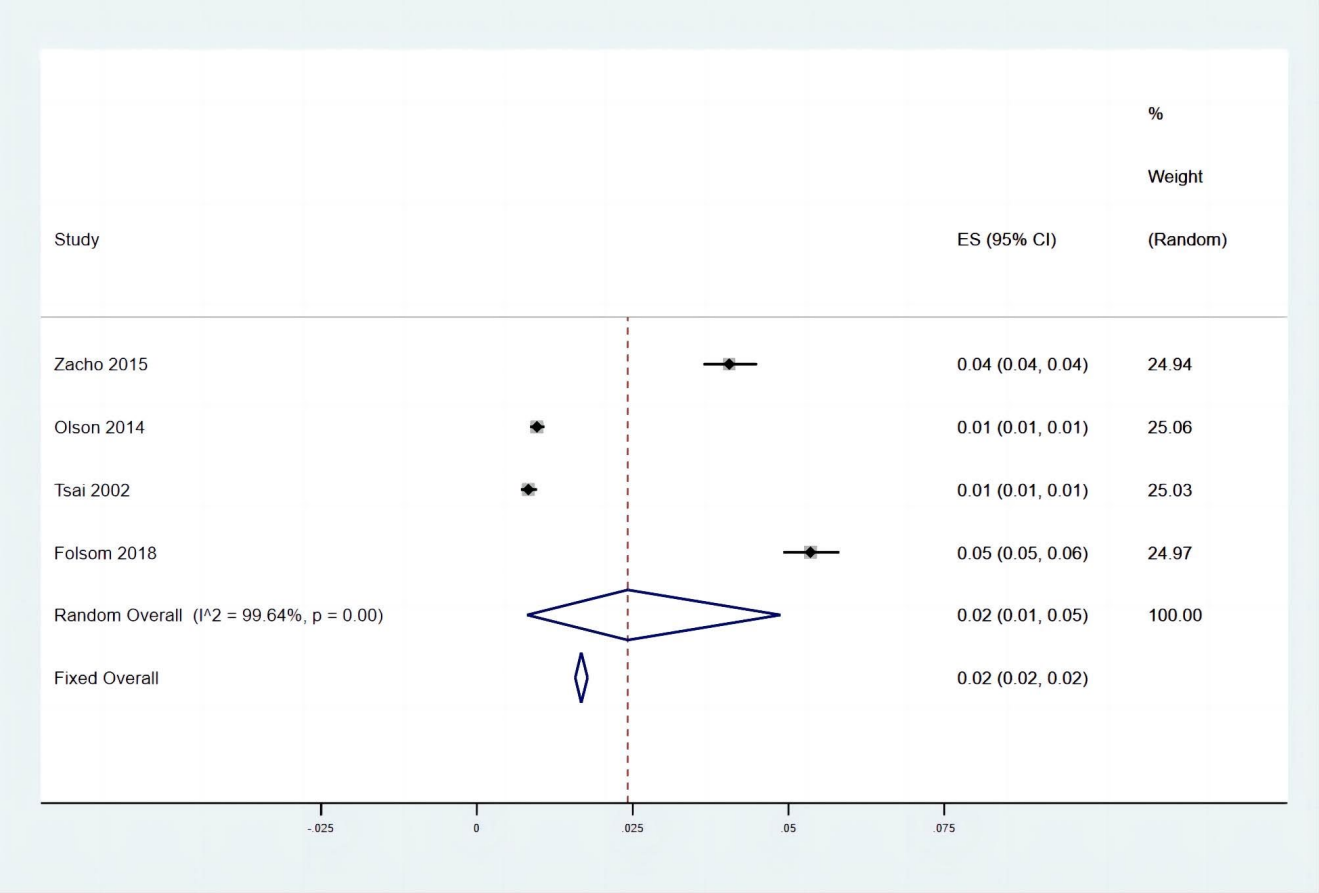
The pooled incidence rate of VTE in general populations. A total of 65,162 general participants involved in this part, of whom 1289 (2%, 95%CI, 1—5%) developed a VTE during a long-time observation

There was a total of 65,162 general participants involved in this part, of whom 1289 (2%, 95%CI, 1—5%) developed a VTE during a long-time observation (Fig. 1) [13–16]. A total of four indices were included in the analysis, in which hs-CRP (MD, 0.63, 95%CI, 0.21—1.05) and the percentage of people with CRP > 3ug/ml (OR, 1.52, 95%CI, 1.18—1.96) obtained statistical significance between VTE patients and health controls, indicating that higher levels of hs-CRP and CRP > 3ug/ml were associated with the development of VTE in general populations (Table 2). A funnel plot is shown in Figure S2.

**Table 2:** Pool analysis of association between inflammatory markers and VTE

Inflammatory markers	Involved studies	Num. of cases (VTE vs. Health control)	MD, 95%CI
Part I			
WBC, ×10^9^/L	Olson, 2014; Tsai, 2002	427 vs. 46,617	−0.05,−0.23—0.14
CRP, ug/ml	Tsai, 2002	89 vs. 307	−0.40,−1.23—0.43
hs-CRP^*^, ug/ml	Olson, 2014	268 vs. 27,539	0.63, 0.21—1.05
CRP > 3ug/ml	Olson, 2014; Zacho, 2010; Folsom, 2018	459/16,108 vs. 671/29,817^#^	1.39, 1.23—1.57^ξ^
Part II			
WBC, ×10^9^/L	Rattazzi, 2013; Yang, 2015; Artoni, 2018	902 vs. 636	0.32,−0.22—0.85
Neutrophil, ×10^9^/L	Rattazzi, 2013; Artoni, 2018	826 vs. 539	0.20,−0.62—1.02
Lymphocyte, ×10^9^/L	Rattazzi, 2013; Yang, 2015; Artoni, 2018	902 vs. 636	−0.01,−0.07—0.06
Monocyte, ×10^9^/L	Rattazzi, 2013	240 vs. 240	0.03, 0.00—0.05
PLR	Artoni, 2018	586 vs. 299	−4.74,−11.02—1.54
NLR	Artoni, 2018	586 vs. 299	−0.10,−0.23—0.03
hs-CRP^*^, ug/ml	Rattazzi, 2013; Vormittag, 2005; Matos, 2011; Luxembourg, 2009	674 vs. 672	0.85, 0.61—1.08
CRP, ug/ml	Artoni, 2018	586 vs. 299	0.66, 0.20—1.13
IL−6^*^, pg/ml	Poredos, 2011; Matos, 2011	168 vs. 174	0.47, 0.25—0.70
Part III			
WBC, ×10^9^/L	Farah, 2019; Bakirci, 2015; Reiter, 2003; Zakai, 2013; Ming, 2018; KAMIŞLI, 2012; Akboga, 2017; Zakai, 2004; Tekeşin, 2019	1016 vs. 1245	1.43, 0.88—1.98
Neutrophil, ×10^9^/L	Farah, 2019; Ming, 2018; Kurtipek, 2015; KAMIŞLI, 2012; Akboga, 2017; Tekeşin, 2019	609 vs. 480	1.79, 1.02—2.56
Lymphocyte, ×10^9^/L	Farah, 2019; Ming, 2018; Kurtipek, 2015; KAMIŞLI, 2012; Akboga, 2017; Tekeşin, 2019	609 vs. 480	−0.23,−0.35—−0.12
Monocyte, ×10^9^/L	Ming, 2018; Tekeşin, 2019	151 vs. 145	0.17, 0.14—0.21
PLR	Farah, 2019; Ming, 2018; Kurtipek, 2015; Akboga, 2017; Tekeşin, 2019	574 vs. 453	33.11, 24.45—41.78
NLR	Farah, 2019; Bakirci, 2015; Ming, 2018; Kurtipek, 2015; KAMIŞLI, 2012; Akboga, 2017; Wang 2018; Tekeşin, 2019	781 vs. 555	1.34, 0.95—1.73
hs-CRP, ug/ml	Bakirci, 2015; Zhou, 2015; Wang 2018; Tekeşin, 2019	276 vs. 197	3.72, 1.45—5.99
CRP, ug/ml	Reiter, 2003; Ramacciotti, 2011; Lowe, 2000	148 vs. 279	1.05,−0.79—2.88
IL−6, pg/ml	Wang 2018	95 vs. 41	5.99, 4.52—7.46

^*^transferred from median(IQR). ^#^num. of patients with VTE/CRP > 3ug/ml vs. VTE/CRP < 3ug/ml. ^ξ^OR, 95%CI.

### Part II: inflammatory markers in patients with previous VTE

**Fig. 2:**
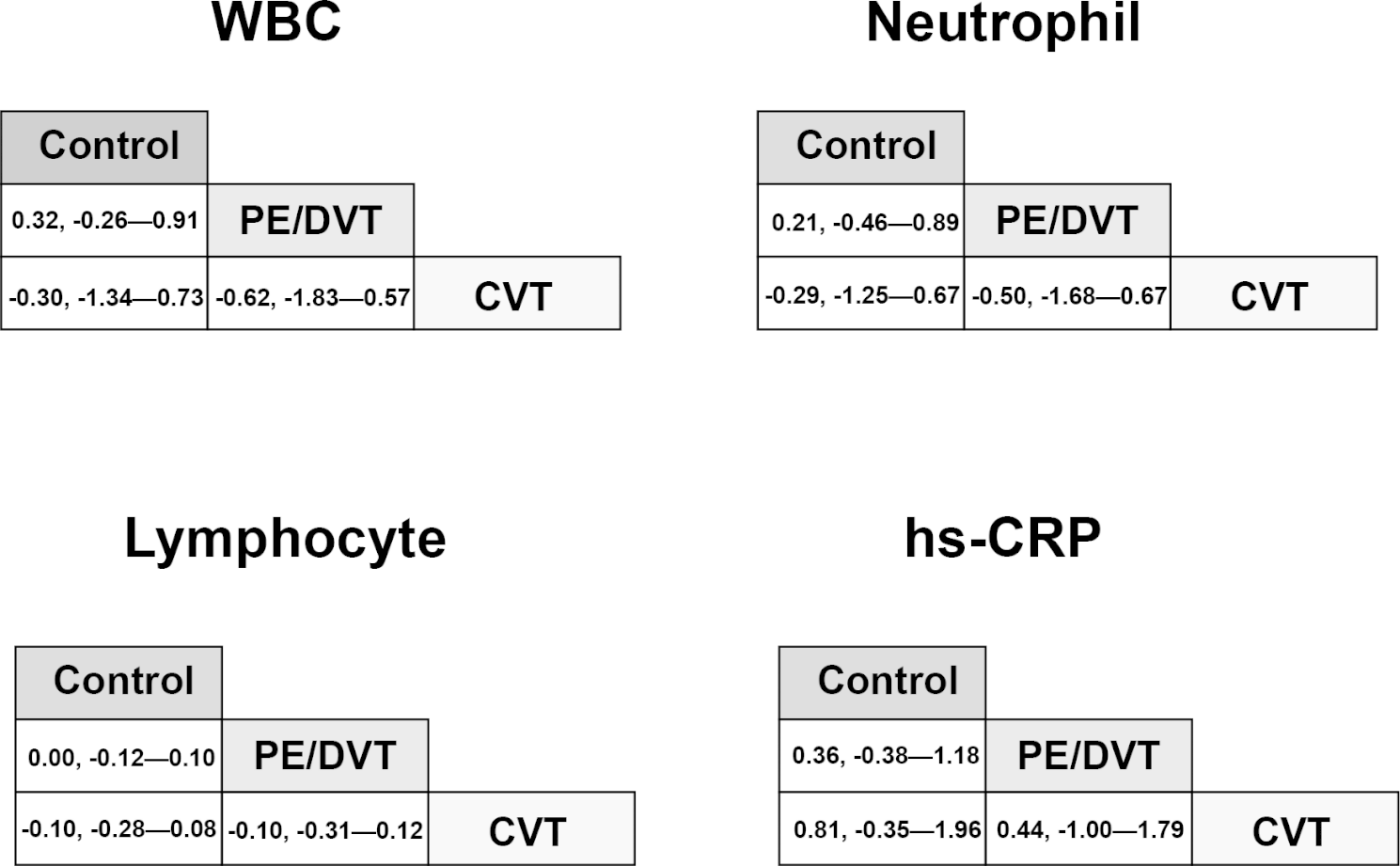
The network meta-analysis for pair-comparisons of inflammatory biomarkers among the cohorts with previous PE/DVT, CVT and controls. As for the levels of WBC, neutrophil, lymphocyte and hs-CRP, we did not find significant differences among these cohorts

A total of 2501 cases were involved in this part, of which 1385 cases had previous VTE and 1116 cases did not have VTE [17–23]. In this study, nine indices were measured in which monocytes (MD, 0.03, 95%CI, 0.00—0.05), hs-CRP (MD, 0.85, 95%CI, 0.61—1.08), CRP (MD, 0.66, 95%CI, 0.20—1.13) and IL−6 (MD, 0.47, 95%CI, 0.25—0.70) showed significant differences between VTE cases and those without VTE (Table 2). A funnel plot evaluating publication bias is shown in Figure S3. Pair comparisons conducted by network meta-analysis did not find significant differences among cohorts with previous PE/DVT, CVT and controls (Fig. 2).

### Part III: inflammatory biomarkers in newly diagnosed VTE patients

**Fig. 3:**
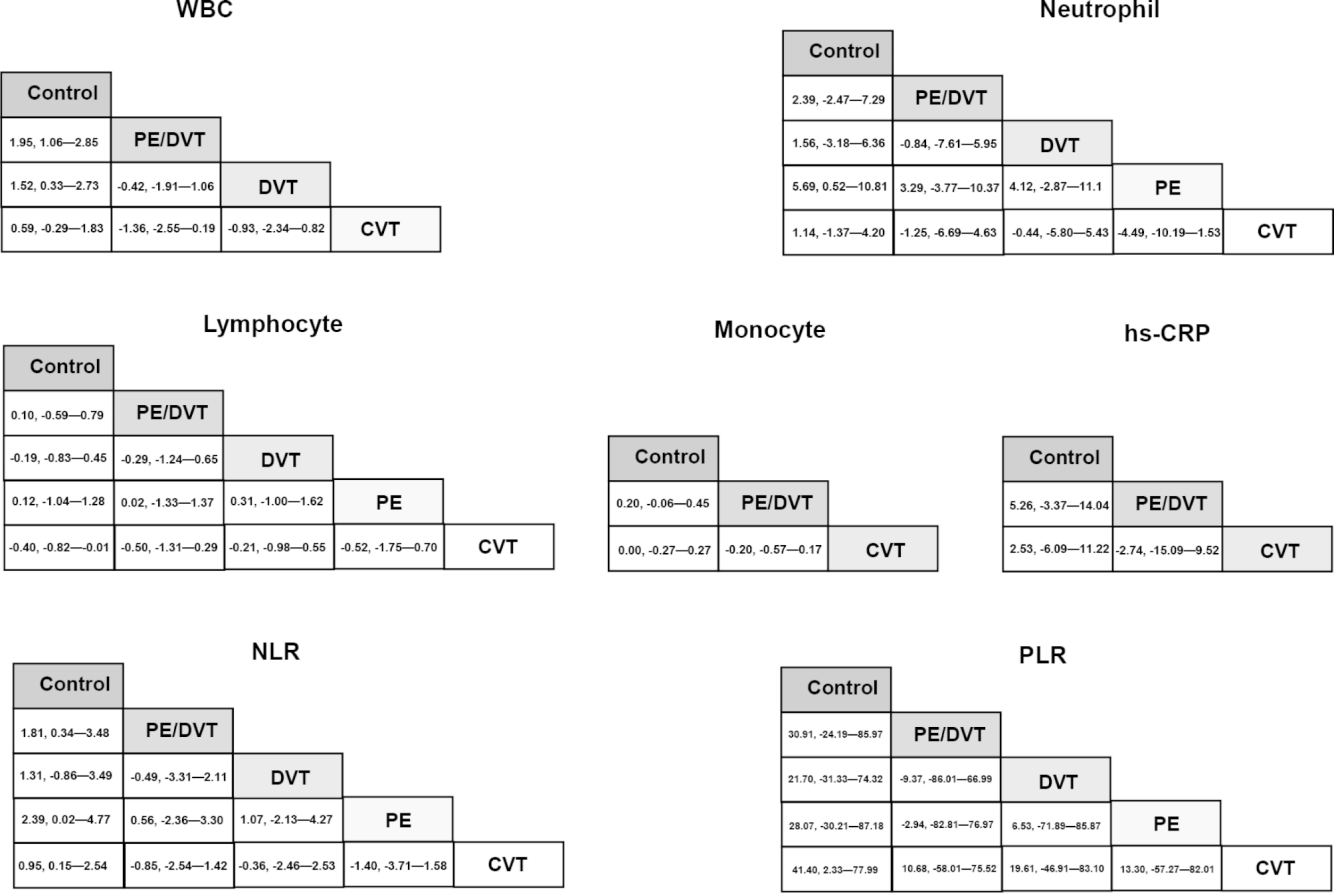
The network meta-analysis for pair-comparisons of inflammatory biomarkers among the cohorts with current PE/DVT, PE, DVT, CVT and controls. As for the levels of WBC, neutrophil, lymphocyte, monocyte, hs-CRP, NLR and PLR, the pair comparison between cohorts with PE/DVT, PE, DVT and CVT reached null hypothesis. When comparing with the controls, the cohort with PE/DVT had significant differences in the levels of WBC (MD, 1.95, 95%CI, 1.06—2.85) and NLR (MD, 1.81, 95%CI, 0.34—3.48); DVT had significant differences in the levels of WBC (MD, 1.52, 95%CI, 0.33—2.73); PE had significant differences in the levels of neutrophil (MD, 5.69, 95%CI, 0.52—10.81) and NLR(MD, 2.39, 95%CI, 0.02—4.77); CVT had significant differences in the levels of lymphocyte (MD,−0.40, 95%CI,−0.82—−0.01), NLR (MD, 0.95, 95%CI, 0.15—2.54) and PLR (MD, 41.40, 95%CI, 2.33—77.99)

A total of 3053 cases were enrolled in this part, of which 1331 cases were newly diagnosed with VTE, and 1722 cases were without VTE or healthy persons [24–35, 37, 39]. There were nine indices compared between these two populations. Compared to control persons, the pooled analysis (Table 2) found that patients with VTE had different levels of WBC (MD, 1.43, 95%CI, 0.88—1.98), neutrophil (MD, 1.79, 95%CI, 1.02—2.56), lymphocyte (MD,−0.23, 95%CI,−0.35—−0.12), monocyte (MD, 0.17, 95%CI, 0.14—0.21), PLR (MD, 33.1, 95%CI, 24.45—41.78), NLR (MD, 1.34, 95%CI, 0.95—1.73), hs-CRP (MD, 3.72, 95%CI, 1.45—5.99) and IL−6 (MD, 5.99, 95%CI, 4.52—7.46). A funnel plot is presented in Figure S4. We performed a network meta-analysis for pair-comparison among the cohorts with respect to PE/DVT, DVT, PE, CVT, and health. Compared to health controls, patients with PE/DVT had higher levels of WBC (MD, 1.946, 95%CI, 1.063—2.846) and NLR (MD, 1.81, 95%CI, 0.34—3.483), those with DVT had higher levels of WBC (MD, 1.521, 95%CI, 0.3316—2.732), those with PE had higher neutrophil levels (MD, 5.685, 95%CI, 0.5205—10.81) and NLR (MD, 2.385, 95%CI, 0.01506—4.77), and those with CVT had higher levels of NLR (MD, 0.9545, 95%CI, 0.1529—2.535) and PLR (MD, 41.4, 95%CI, 2.327—77.99), and lower lymphocyte levels (MD, 0.3988, 95%CI, 0.005852—0.8154). The pair comparison between cohorts with PE/DVT, PE, DVT and CVT reached null hypothesis. (Fig. 3).

Given the high heterogeneity between the involved studies, sensitivity analysis was performed by removing the studies without a definitive time for blood sample collection or diagnosis of VTE (Model 1), and the studies using the median (IQR) for analysis (Model 2). In Model 1, there were higher levels of WBC (MD, 1.55, 95%CI, 0.97—2.12), platelet (MD, 15.83, 95%CI, 6.64—25.03), neutrophil (MD, 2.09, 95%CI, 1.03—3.14), PLR (MD, 26.26, 95%CI, 15.67—36.85), NLR (MD, 1.41, 95%CI, 0.93—1.88) and hs-CRP (MD, 1.80, 95%CI, 0.46—3.15) in patients diagnosed with VTE compared to health controls. Additional network meta-analysis showed that patients with PE/DVT had a higher level of WBC (MD, 1.947, 95%CI, 0.8287—3.147) than health controls (Figure S5). In Model 2, higher levels of WBC (MD, 1.68, 95%CI, 1.22—2.15), platelet (MD, 22.66, 95%CI, 8.92—36.41), neutrophil (MD, 2.10, 95%CI, 1.31—2.89), PLR (MD, 33.22, 95%CI, 23.90—42.54) and NLR (MD, 1.59, 95%CI, 1.22—1.96), and lower levels of lymphocyte (MD,−0.19, 95%CI,−0.31—−0.06) could be observed in patients with VTE compared to health controls. Additional network meta-analysis showed that patients with PE/DVT had higher levels of WBC (MD, 2.066, 95%CI, 1.106—3.406) and platelet (MD, 20.47, 95%CI, 1.063—42.66), those with DVT had a higher level of WBC (MD, 1.518, 95%CI, 0.1823—2.903), and those with CVT had a higher levels of platelets (MD, 52.1, 95%CI, 8.826—95.16), compared to health controls (Figure S6).

## Discussion

A large pool of studies has investigated the association of inflammatory biomarkers with VTE till now, and only 25 articles were recruited in this study after screening [13–35, 37, 39]. To our knowledge, this is the first meta-analysis to establish the link between inflammatory biomarkers and VTE, which comprises PE, DVT and CVT. Our results revealed that inflammatory biomarkers may not only be associated with an increased risk of the occurrence of VTE but also serve as an auxiliary method for diagnosing VTE in clinical settings. However, given the inevitable heterogeneity, unclear methodologies, and data transformation, the results should be interpreted with caution.

Inflammatory processes may increase the risk of VTE, because a procoagulant state may be induced by a series of pro-inflammatory factors [3]. Previous studies indicated that CRP > 3ug/ml may predict the occurrence of VTE in the future [13, 14, 16]. Our pooling results in Part I, which included 3 studies, further corroborated this point. CRP can promote P-selectin expression, increase tissue factor expression, decrease prostacyclin release, and improve cyclooxygenase-2, all of which can stimulate or enhance platelet adhesion and responsiveness [40–42]. Although an elevated CRP level is robustly related to the risk of VTE, this may not necessarily be a causal association, as demonstrated by the absence of association between genetically elevated CRP and the risk of VTE [36]. The increased level of hs-CRP and low platelet level also predicted VTE occurrence; however, these were not pooled results [14].

In terms of patients with previous VTE, the levels of hs-CRP, CRP, IL-6, and monocyte were elevated, but other important biomarkers such as NLR, PLR, neutrophil, lymphocyte and WBC were close to normal. The network meta-analysis did not find conspicuous differences in inflammatory biomarkers among populations with previous PE/DVT, CVT and health issues. These results indicated that VTE was also characterized by a mild inflammatory response in the long run. CRP, especially hs-CRP, not only predicts the occurrence of VTE mentioned above, but also directly reflects the inflammatory response for VTE in the chronic stage. IL-6 is the most important cytokine in thrombus formation and progression, demonstrated by inducing the expression of tissue factor, fibrinogen, factor VIII, and von Willebrand factor to promote coagulation, and lowering the concentration of anthrombin, protein S, and thrombomodulin to inhibit anticoagulation [43, 44]. Monocytes are important cells for VTE resolution, as they synthesize chemokines, cytokines, proteases and protease receptors that can amplify thrombus extension and conversely may promote clot retraction and resolution [9]. However, the sample sizes for the association of VTE with IL-6 and monocyte were too small to draw a convincing conclusion currently. We considered that these elevated inflammatory biomarkers at the chronic stage of VTE may represent the mild activity of thrombus development.

A substantial inflammatory response could be seen in patients with newly diagnosed VTE. Consistent with other systemic inflammatory diseases such as atherosclerosis, our results showed that there were obviously a variety of biomarkers that increased in these patients, including WBC, neutrophil, monocyte, PLR, NLR, hs-CRP, and IL-6, and in addition, the level of lymphocyte decreased [45]. This was in line with the finding of profoundly increased levels of inflammatory biomarkers around arterial thrombosis, especially for PLR, NLR, and IL-6 [46]. We hypothesized that arterial thrombosis features inflammatory responses similar to venous thrombosis, but arterial thrombosis evolves in a different manner. The relevant evidence was very poor, so a large sample-size observational study or secondary network meta-analysis is needed to identify the discrepancies between them in the future. Subsequent network meta-analysis presented that WBC and NLR levels in patients with PE/DVT, WBC in DVT, neutrophil and NLR in PE and NLR and PLR in CVT were significantly higher, and lymphocytes in CVT were substantially lower than in health controls. Although the inflammatory characteristics of each type of VTE were different compared to health controls, the pair comparison did not draw a statistical difference between themselves. These results indicated that the inflammatory response might not be appropriate to distinguish PE, DVT, and CVT, but might have a high value for VTE diagnosis. To diminish the heterogeneity among the involved studies, a sensitivity analysis was performed with caution. Studies without a definite measurement time were ruled out of Part III because no evidence verified that the patients were newly diagnosed with VTE when obtaining the blood samples. The pooling results of the remaining studies (Model 1) showed that the levels of WBC, neutrophil, PLR, NLR and hs-CRP were substantially higher in VTE patients than in health controls. Furthermore, studies containing the data needed to converse the median (IQR) to the mean (SD) were removed from Part III because this data transformation may bias the results towards null hypothesis, despite a relatively stable algorithm provided by Luo et al. and Wan et al. [47, 48] The combined results of the remaining studies (Model 2) presented higher levels of WBC, neutrophils, PLR, and NLR, and a lower level of lymphocytes could be seen in patients with VTE compared to health controls. The levels of WBC, neutrophil, PLR, and NLR maintained significant differences between groups regardless of whether they underwent sensitivity analysis or not.

Neutrophil is considered responsible for the inflammatory response in thrombus formation, which plays a double-edged sword role during thrombosis: massive activation in the early stage causes tissue damage and thrombus propagation; while late activation leads to resolution of the thrombus [49]. An elevated level of neutrophils can be commonly seen in both arterial and venous thrombosis events. NETs extruded from neutrophils are involved in microvascular thrombosis, coined as ‘immunothrombosis’, which can trigger fibrin formation to trap and destroy invading microorganisms [8]. In contrast, the level of lymphocyte is always restrained with thrombus formation, however, the definite mechanisms remain unclear. Elevated neutrophils and decreased lymphocytes result in an increase in NLR and PLR. NLR and PLR are considered the conspicuous index of cerebral arterial ischemia and might be more accurate for the diagnosis of thrombus than immune cells [50]. They are also applied to identify CVT diagnosis and prognosis in some research studies. The diagnostic value of NLR might be more conspicuous, because it still obviously increased in patients with PE/DVT, PE and CVT when undergoing network meta-analysis.

There were several limitations in this study. First, number of involved studies was not large enough to draw a comprehensive conclusion (such as IL-6 and monocyte), especially when conducting a network meta-analysis. Therefore, the pair-comparison could not obtain a convincing result and only served as additional evidence in the current study. Second, although PE, DVT and CVT have some different formation mechanisms, the patients with CVT, PE, or DVT were grouped as VTE in this study. This might render our results underpowered. However, we considered the main pathogenesis of both CVT and PE/DVT is hypercoagulable state, and the current and previous thrombus might affect the body’s inflammatory response generally. In order to reach a more convincing conclusion, we conducted pair-wise comparisons among the patients with PE/DVT, PE, DVT and CVT through net-work meta-analysis. Third, despite the fact that we performed a sensitivity analysis in this study, the different study design and data collection time between the involved studies produced the inevitable bias that could have impacted our conclusion. Finally, the level of some markers (such as hs-CRP and CRP), markers expressed as median (IQR), was conversed to mean (SD) for the pool analysis. Although the conversion process was well-accepted, the pooling analysis might not be available to the data, not following the Gaussian distribution and can create bias in the results, leading to a null hypothesis.

## Conclusions

This study provided a secondary analysis for the association between inflammatory biomarkers and VTE, and found that inflammatory biomarkers including hs-CRP and CRP might be regarded as risk factors for future VTE occurrence; elevated levels of monocyte, hs-CRP, CRP, and IL-6 might represent the previous VTE; a series of markers such as WBC, neutrophil, PLR and NLR increased during the acute phase of VTE. In general, inflammatory biomarkers may not only be correlated with an increased risk of VTE, but may also give a hint of the occurrence of VTE in clinical settings. In light of the limitations of the study, further larger epidemiologic studies and secondary analyses are warranted to reach a more convincing conclusion.

## Electronic supplementary material

Below is the link to the electronic supplementary material.

Supplementary Material 1

## Data Availability

The data is available from the corresponding author on reasonable requests.

## Abbreviations

VTE: venous thromboembolism
NETs: neutrophils extrude neutrophil extracellular traps
FXII: factor XII
vWF: von willebrand factor
TF: tissue factor
CVT: cerebral venous thrombosis
PE: pulmonary embolism
DVT: deep vein thrombosis
SD: standard deviate
IQR: interquartile range
OR: odds ratio
RR: relative risk
WBC: white blood cells
PLR: platelet-lymphocyte ratio
NLR: neutrophil-lymphocyte ratio
hs-CRP: high-sensitivity C-reactive protein
CRP: C-reactive protein
IL-6: interleukin-6
