# Supplementary material for: Association between inflammatory biomarkers and venous thromboembolism: a systematic review and meta-analysis
Source: Thromb J. 2023 Jul 31;21:82. doi: 10.1186/s12959-023-00526-y (PMC10388478; doi:10.1186/s12959-023-00526-y)
Supplement: Supplementary file 1 — Supplementary Material 1 [file 12959_2023_526_MOESM1_ESM.pdf]

Supplementary Material

Table S1: Search strategies and results

Database 1: Pubmed
Search date: 2023:4:1
Search Strategy:
(("venous thrombolism"[Title] OR "venous thromboembolism"[Title] OR "venous thrombosis"[Title] OR "venous embolism"[Title] OR "vein embolism"[Title] OR "pulmonary embolism"[Title] OR "pulmonary thromboembolism"[Title] OR "Venous Thrombosis"[MeSH Terms]) AND ("inflammation"[MeSH Terms] OR "inflammat*"[Title] OR "immun*"[Title] OR "neutrophil"[Title] OR "lymphocyte"[Title] OR "monocyte"[Title] OR "c reaction protein"[Title] OR "c-reaction protein"[Title] OR "high-sensitivity-c-reaction protein"[Title] OR "leukocyte"[Title] OR "NLR"[Title] OR "LMR"[Title] OR "PLR"[Title] OR "white blood cell"[Title])) AND ("humans"[Filter])

Database 2: Embase
Search date: 2023:4:1
Search Strategy:
((venous thrombolism:ti OR venous thromboembolism:ti OR venous thrombosis:ti OR venous embolism:ti OR vein embolism:ti OR pulmonary embolism:ti OR pulmonary thromboembolism:ti OR Vein Thrombosis/exp) AND (inflammation/exp OR inflammat*:ti OR immun*:ti OR neutrophil:ti OR lymphocyte:ti OR monocyte:ti OR c reaction protein:ti OR c-reaction protein:ti OR high-sensitivity-c-reaction protein:ti OR leukocyte:ti OR NLR:ti OR LMR:ti OR PLR:ti OR white blood cell:ti)) AND (human/exp OR human:ti OR patient:ti OR patients:ti)

Figure S1

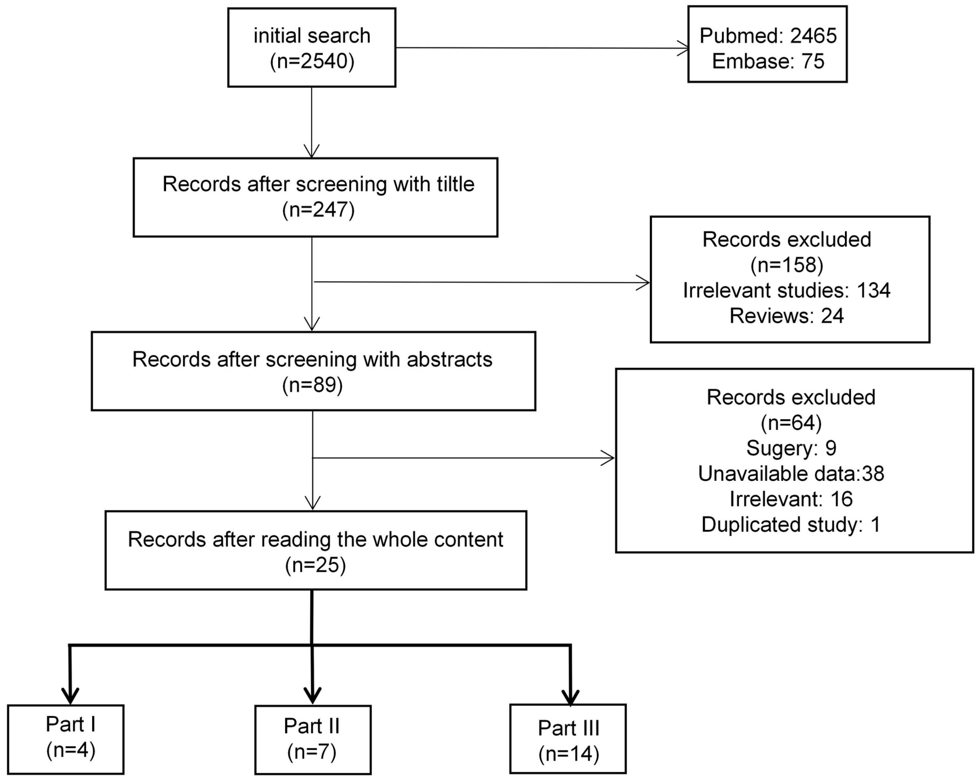

Figure S1. Study selection flowchart. The search strategy identified 247 articles with adopted title published before April 2023. During the subsequent screening process, there were 134 irrelevant studies and 24 reviews excluded by screening abstracts. After thorough reading of the entire content, 25 articles that followed our study selection criteria remained in this study. None of the articles were involved after reviewing the references of the retrieved articles. There were 4 articles in Part I, 7 articles in Part II, 14 articles in Part III.

Figure S2

Part I

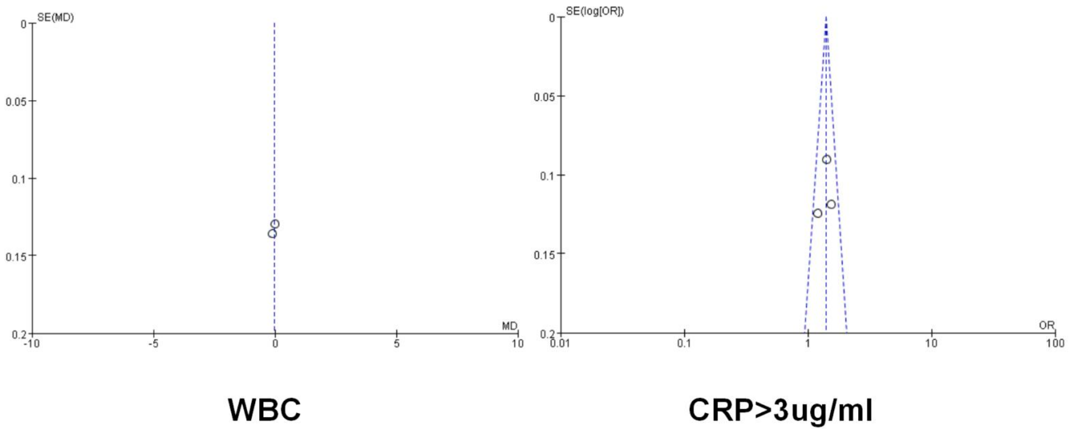

Figure S2. Publication bias from the included articles about the inflammatory markers predicting VTE occurrence. The graph is symmetrical, which demonstrates that the publication bias of included studies is low.

Figure S3

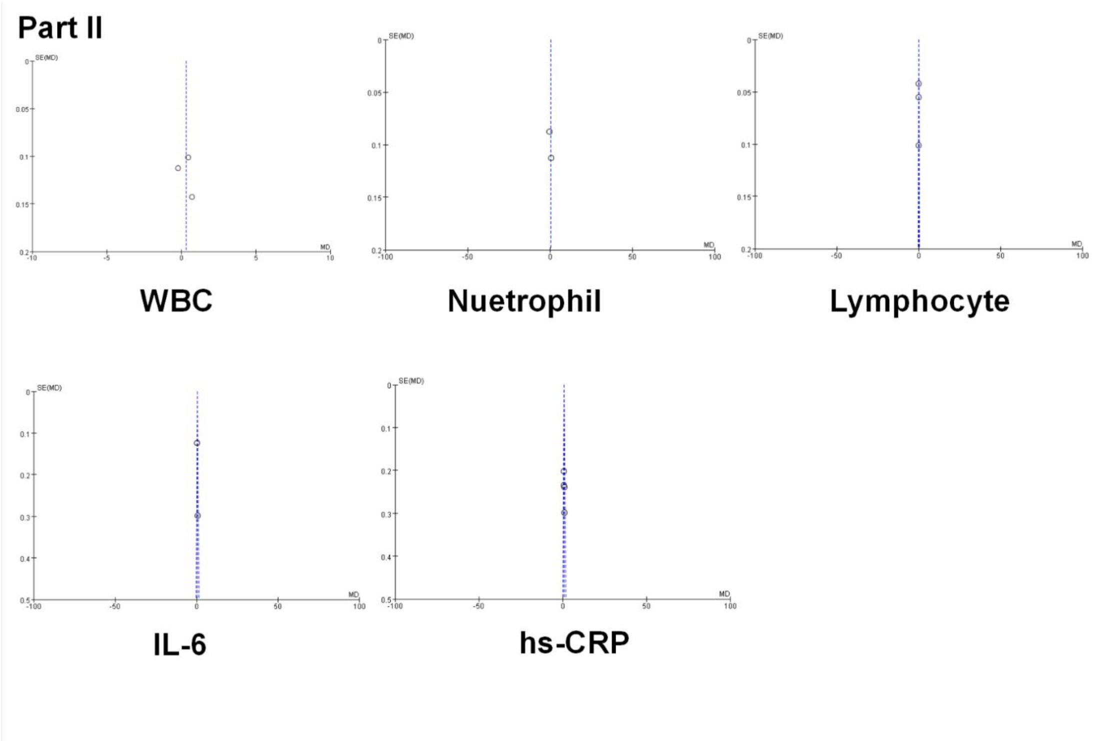

Figure S3. Publication bias from the included articles about the association between the inflammatory markers and previous VTE. The publication bias of these involved studies is low according to the symmetrical graphs.

Figure S4

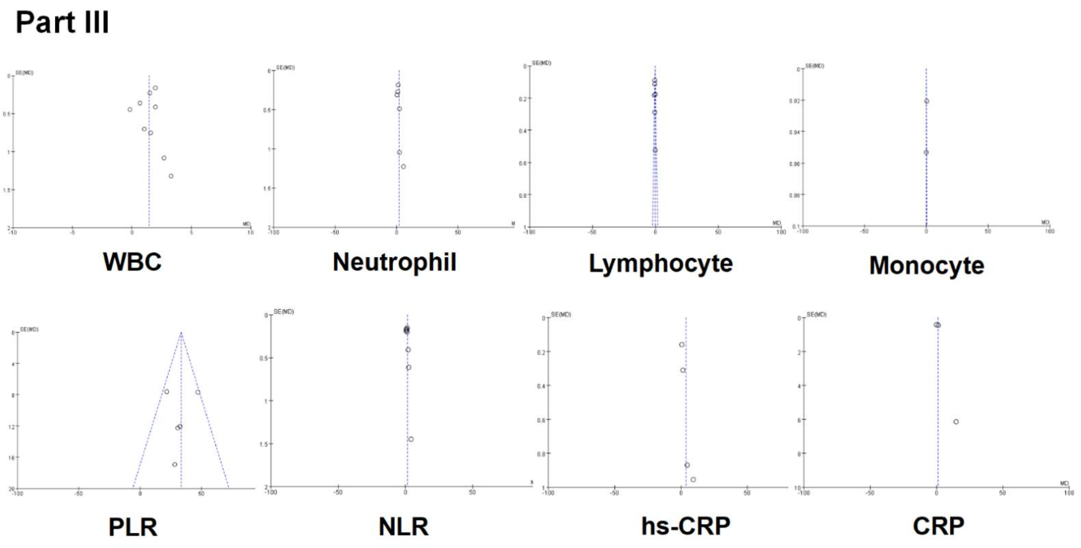

Figure S4. Publication bias from the included articles about the association between the inflammatory markers and newly diagnosed VTE. The symmetrical graphs indicates the publication bias of the involved studies is low, except for WBC.

Figure S5

WBC

Control			
1.95, 0.83—3.15	PE/DVT		
1.52, -0.03—3.11	-0.42, -2.41—1.53	DVT	
0.55, -0.85—2.71	-1.41, -3.12—1.05	-1.00, -2.93—1.79	CVT

Neutrophil

Control				
2.41, -4.18—8.96	PE/DVT			
1.56, -4.88—8.17	-0.83, -10.04—8.51	DVT		
5.70, -1.14—12.45	3.29, -6.17—12.82	4.14, -5.38—13.40	PE	
1.26, -3.13—6.30	-1.17, -8.98—7.33	-0.34, -8.13—7.90	-4.41, -12.37—4.16	CVT

Lymphocyte

Control				
0.10, -0.66—0.88	PE/DVT			
-0.19, -0.92—0.54	-0.29, -1.34—0.77	DVT		
0.13, -1.10—1.32	0.03, -1.41—1.42	0.32, -1.09—1.69	PE	
-0.50, -1.05—-0.10	-0.60, -1.53—0.37	-0.31, -1.20—0.64	-0.62, -1.92—0.73	CVT

hs-CRP

Control		
1.33, -4.93—7.53	PE/DVT	
2.37, -1.99—7.03	1.05, -6.56—8.86	CVT

NLR

Control				
1.80, -0.38—4.19	PE/DVT			
1.31, -1.90—4.51	-0.49, -4.50—3.37	DVT		
2.39, -0.98—5.74	0.56, -3.53—4.53	1.08, -3.60—5.66	PE	
0.86, -0.39—3.55	-0.94, -3.29—2.69	-0.47, -3.45—4.05	-1.45, -4.73—3.07	CVT

PLR

Control				
30.88, -15.86—77.02	PE/DVT			
21.49, -21.78—64.17	-9.42, -73.01—53.18	DVT		
28.39, -22.4—79.22	-2.45, -70.79—66.61	7.14, -59.34—73.62	PE	
32.61, -13.71—78.92	1.78, -63.69—67.28	11.31, -51.81—74.05	4.06, -64.23—73.15	CVT

Figure S5. The network meta-analysis for pair-comparisons of inflammatory biomarkers among the cohorts with current PE/DVT, PE, DVT, CVT and controls conducted in Model 1. As for the levels of WBC, neutrophil, lymphocyte, hs-CRP, NLR and PLR, the pair comparison between cohorts with PE/DVT, PE, DVT and CVT reached null hypothesis. When comparing with control, the cohort with PE/DVT had significant differences in the levels of WBC (MD, 1.95, 95%CI, 0.83—3.16); CVT had significant differences in the levels of lymphocyte (MD, -0.50, 95%CI, -1.05—-0.10).

Figure S6

WBC

Control			
2.07, 1.11—3.41	PE/DVT		
1.52, 0.18—2.90	-0.55, -2.49—1.07	DVT	
1.06, -0.12—2.88	-1.03, -2.69—1.01	-0.47, -2.19—1.87	CVT

Neutrophil

Control				
2.40, -3.76—8.50	PE/DVT			
1.56, -4.55—7.69	-0.83, -9.45—7.74	DVT		
5.71, -0.71—11.94	3.31, -5.50—12.11	4.15, -4.65—12.83	PE	
1.62, -2.48—6.29	-0.77, -8.01—7.04	0.04, -7.17—7.84	-4.02, -11.38—3.92	CVT

Lymphocyte

Control				
0.10, -0.38—0.58	PE/DVT			
-0.19, -0.57—0.20	-0.29, -0.91—0.32	DVT		
0.13, -0.97—1.27	0.03, -1.16—1.26	0.32, -0.83—1.51	PE	
-0.30, -0.64—-0.04	-0.40, -0.98—0.18	-0.11, -0.62—0.40	-0.43, -1.60—0.72	CVT

NLR

Control				
1.84, -1.10—4.90	PE/DVT			
1.31, -2.98—5.57	-0.53, -5.79—4.57	DVT		
2.38, -1.94—6.75	0.53, -4.77—5.78	1.07, -4.98—7.23	PE	
1.82, -0.76—5.68	-0.08, -3.83—5.00	0.45, -4.12—6.44	-0.55, -5.30—5.46	CVT

PLR

Control				
30.53, -32.17—93.48	PE/DVT			
21.40, -38.33—81.69	-9.17, -96.11—78.36	DVT		
28.74, -37.11—93.69	-1.79, -92.87—88.39	7.24, -82.26—95.19	PE	
46.90, -13.32—106.10	16.37, -70.09—102.70	25.39, -59.58—110.20	18.20, -70.43—107.20	CVT

Figure S6. The network meta-analysis for pair-comparisons of inflammatory biomarkers among the cohorts with current PE/DVT, PE, DVT, CVT and controls conducted in Model 2. As for the levels of WBC, neutrophil, lymphocyte, NLR and PLR, the pair comparison between cohorts with PE/DVT, PE, DVT and CVT reached null hypothesis. When comparing with control, the cohort with PE/DVT had significant differences in the levels of WBC (MD, 2.07, 95%CI, 1.11—3.41); DVT had significant differences in the levels of WBC (MD, 1.52, 95%CI, 0.18—2.90); CVT had significant differences in the levels of lymphocyte (MD, -0.30, 95%CI, -0.64—-0.04).
